# An Account of a Remarkable Operation on a Broken Arm

**Published:** 1781-07

**Authors:** 


II. *An account of a remarkable operation on a broken arm. By Mr.* Edward Ford, *Surgeon to the Weftminfter General Difpenfary. Communicated by* Samuel Foart Simmons, M. D. F. R. S. *Read* July 2, 1781.

THOMAS SMITH, a native of Ireland, aged thirty-five years, being at Milford Haven, in October 1775, on his way to Ireland, had the misfortune to break his arm. It was immediately bound up by a furgeon of that place, and the day following he failed for Galway, and from thence walked to Limerick by fhort ftages, applying, on the road, to a furgeon, who examined his arm. At Limerick he put himfelf under the care of an eminent practitioner, who continued the fame or fimilar bandages. All this time it does not appear by his narrative that any any remarkable circumftances occurred. Neverthelefs at the end of fix weeks the fractured ends of the bone were not united. He was then advifed to have cold water pumped upon his arm.—This plan was purfued for fome time.

He now returned to England, and applied to various gentlemen at different hofpitals, who tried many topical applications, and even internal medicines, but without effect.

When I faw him, in September 1776, there was a tranfverfe fracture of the radius, about two inches above its juncture with the wrift, the ends of the bone evidently rubbing againft each other. There was no tenfion or fwelling of his arm, although he was in the conftant exercife of his trade, which was that of a fhoemaker.

The inconveniences of which he principally complained were, that he could not perform any labour which required him to grafp his hand; that he was frequently afflicted with cramps in his wrift; and that his arm was quickly tired in the neceffary labour of his bufinefs.

Being perfectly fatisfied that a repetition of the former treatment would be unneceffary, I had at firft propofed to make an incifion down to the fracture, and faw off the ends of the bone, in the manner recommended by Mr. White, and performed performed by him with fuccefs in the Infirmary at Manchefter. But as the patient was not wholly deprived of the ufe of his limb, I thought the rifk would be too great; and confidered, that if nature in the early ftage of the difeafe had been prevented from generating the callus, or the union had been obftructed by any foreign fubftance, the mifchief might probably be remedied merely by cutting down to the fracture. By this means the caufe which prevented the union might be removed, or the offific difpofition excited by the inflammation which would neceffarily refult from this procefs. Accordingly, on the 22d of September, in the prefence of Dr. William Hunter, Dr. Robert Bland, Mr. Vaux, furgeon, Mr. Combe, and feveral other gentlemen, I performed the following operation.

The patient was feated on a chair, and his arm fupported on an inclined plane fixed to the back of that and another chair. The tourniquet being applied, I made a longitudinal incifion about two inches in length, through the integuments on the outfide of the arm, near the courfe of the fupinator radii longus, and of the tendon of the extenfor carpi radialis, both of which being expofed in the diffection, were carefully held afide, whilft I cut down to the fracture. The bone was was covered with its perioſteum which I ſcraped off, by means of a common elaſtic ſpathula, for the ſpace of half an inch above and below the fracture.

I did not find any membrane or other ſubſtance intervening between the fractured parts, but the ends of the bone were rough.

The operation being finiſhed, the wound was filled up with lint, and covered with a pledgit, after which the arm was bound up with an eighteen-tail bandage, and laid upon a pillow in a bent poſition. The patient was imm[illegible]diately put to bed, and an anodyne adminiſtered. The next morning a ſlight ſymptomatic fever coming on, he was bled, and took an opening medicine. After that time he was free from any diſagreeable ſymptom.

In dreſſing his wound for the firſt three weeks, I frequently found upon the lint ſmall exfoliations of the bone, not larger than grains of ſand, but the diſcharge never had that black hue, which uſually attends a caries.

The bone was ſoon covered with granulations, but there was no callus formed till five weeks after the operation, at which period he began to have ſome ſtrength in the limb. At the end of eight weeks I found no kind of crepitation in the bone, and in three months he was entirely well. It is now more than four years ſince the operation was performed, and he continues to have the perfect uſe of his arm.

*Golden-Square,*

*June* 28, 1781.

